# A computational protocol to evaluate the effects of protein mutants in the kinase gatekeeper position on the binding of ATP substrate analogues

**DOI:** 10.1186/s13104-017-2428-9

**Published:** 2017-02-20

**Authors:** Valentina Romano, Tjaart A. P. de Beer, Torsten Schwede

**Affiliations:** 1 Biozentrum, University of Basel, Basel, Switzerland; 2 SIB Swiss Institute of Bioinformatics, Basel, Switzerland

**Keywords:** Computational protein modelling, Protein kinases, Gatekeeper residue, Shokat’s method

## Abstract

**Background:**

The determination of specific kinase substrates in vivo is challenging due to the large number of protein kinases in cells, their substrate specificity overlap, and the lack of highly specific inhibitors. In the late 90s, Shokat and coworkers developed a protein engineering-based method addressing the question of identification of substrates of protein kinases. The approach was based on the mutagenesis of the gatekeeper residue within the binding site of a protein kinase to change the co-substrate specificity from ATP to ATP analogues. One of the challenges in applying this method to other kinase systems is to identify the optimal combination of mutation in the enzyme and chemical derivative such that the ATP analogue acts as substrate for the engineered, but not the native kinase enzyme. In this study, we developed a computational protocol for estimating the effect of mutations at the gatekeeper position on the accessibility of ATP analogues within the binding site of engineered kinases.

**Results:**

We tested the protocol on a dataset of tyrosine and serine/threonine protein kinases from the scientific literature where Shokat’s method was applied and experimental data were available. Our protocol correctly identified gatekeeper residues as the positions to mutate within the binding site of the studied kinase enzymes. Furthermore, the approach well reproduced the experimental data available in literature.

**Conclusions:**

We have presented a computational protocol that scores how different mutations at the gatekeeper position influence the accommodation of various ATP analogues within the binding site of protein kinases. We have assessed our approach on protein kinases from the scientific literature and have verified the ability of the approach to well reproduce the available experimental data and identify suitable combinations of engineered kinases and ATP analogues.

## Background

Phosphorylation is an important mechanism for the post-translational regulation of cellular activity of proteins. The phosphorylation reaction is catalyzed by kinase enzymes by transferring a phosphate group to a specific residue of the protein substrate—typically a serine, threonine or tyrosine—with ATP acting as phosphodonor. Kinases are key regulators for many crucial biochemical pathways, such as the glycogen metabolism [1], cell proliferation, cell division, or apoptosis [2]. The central role of kinases in numerous diseases is extensively documented [3]. For instance the tyrosine protein kinase JAK3 is known being involved in a form of severe combined immunodeficiency [4], the anaplastic lymphoma kinase, ALK, is involved in neuroblastoma development and make ALK an interesting drug target for rationally designed ALK-inhibition therapies for the treatment of human cancers [5]. The identification of the protein substrates of kinase enzymes is therefore of great importance for elucidating their functional role in the cell and to develop disease-specific therapies. However, the identification of specific kinase substrates is highly challenging due to the large number of protein kinases in cells, their substrate specificity overlap and the lack of absolute specificity of inhibitors [6, 7].

**Fig. 1:**
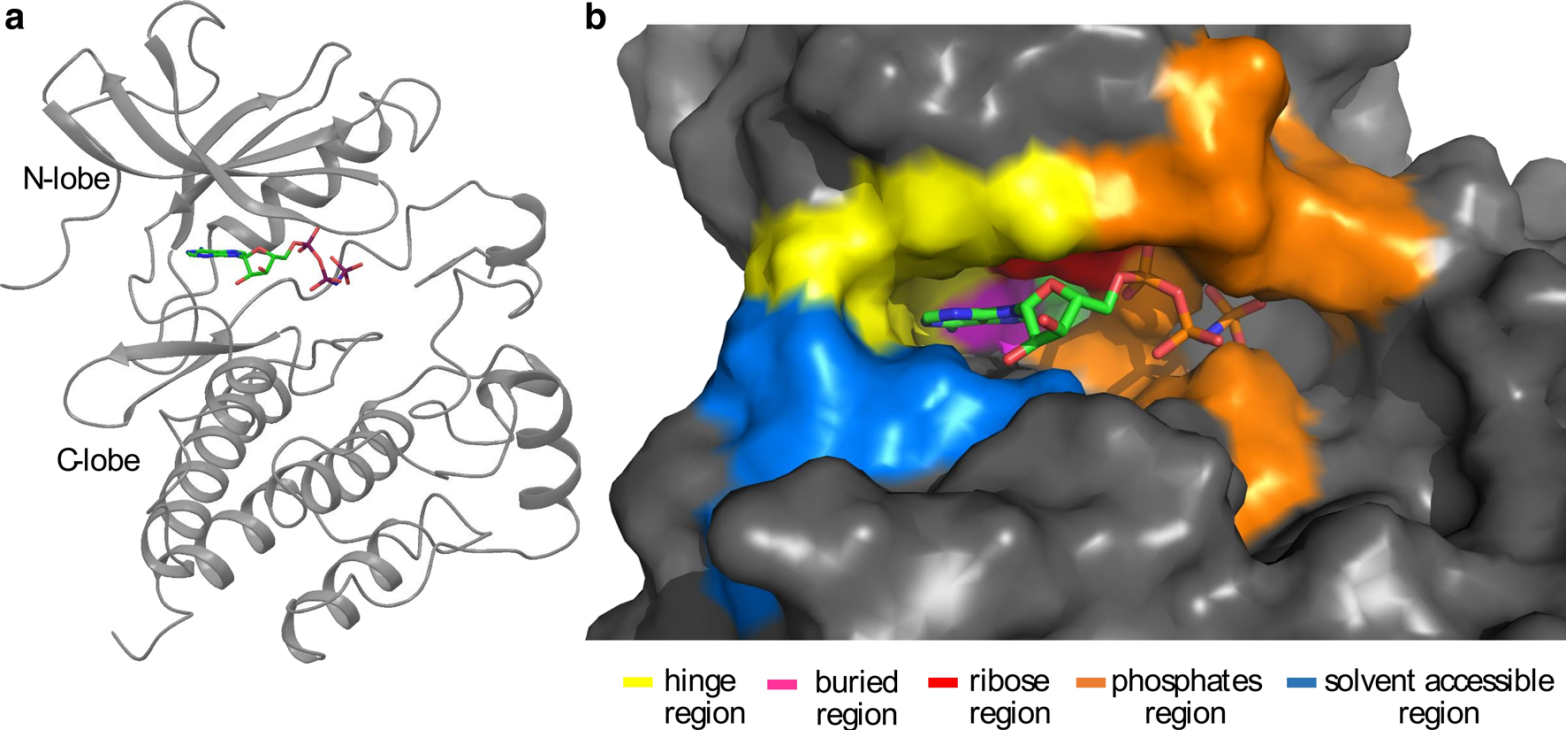
Structure representation of c-Src in complex with ANP (PDB: 2SRC). **a** Ribbon representation of the kinase domain of c-Src in complex with ANP [38]. Tyr belonging to the activation segment is represented as stick. **b** Surface representation of a kinase ligand-binding pocket. The ATP is represented as stick. The five regions belonging to the ATP binding pocket are represented in *different colors* with the buried region behind the ATP

The majority of protein kinases share a bilobal kinase domain fold, where the N-lobe is formed by five β-strands and a single α-helix and the C-lobe is predominantly α-helical [6, 8]. These domains are connected by a short segment called the hinge region [9]. The C-lobe contains the activation segment that is typically composed of 20–30 residues. This lobe is composed of the activation loop that activates protein kinase when a specific residue is phosphorylated (usually a Tyr or a Thr) and the loop that is involved in substrate binding [8] (Fig. 1a). The ATP binding pocket is located in the cleft between the N-lobe and the C-lobe of the kinase domain. It contains a highly conserved Asp which has a significant role in the phosphorylation reaction catalyzed by kinase enzymes. The Asp acts as catalytic base to free up the hydroxyl oxygen of a Ser, Thr or Tyr on the protein substrate. The deprotonated residue is involved in a nucleophilic attack on the terminal (γ) phosphoryl group (${\text{PO}}_{3}^{2 - }$) of ATP [10]. The ATP binding site is made up of five areas, the “adenine region” which corresponds to the hinge region, the “ribose region”, the “phosphates region”, the “solvent accessible region”, and the “buried region” [11, 12] (Fig. 1). The “buried region” is a hydrophobic region located in the back of the ATP pocket and is not occupied by ATP. The size and the shape are controlled by the first amino acid of the hinge region—this amino acid act as a ‘molecular gate’ controlling the accessibility to the buried region. A residue with a small side chain ‘opens the gate’ to the buried region whereas a large side chain effectively ‘closes the gate’ making the buried region inaccessible. For that reason, this residue has been termed the ‘gatekeeper’ residue [13–16] (Fig. 1b). The gatekeeper residue is generally preceded by two hydrophobic residues and followed by an acidic residue and another hydrophobic amino acid. In 73% of human kinases a hydrophobic amino acid with a bulky side chain (Met, Phe or Leu) is observed at that position, 22% have a small residue, such as Thr or Val and the remaining 5% have one of the other amino acids [11, 12, 17, 18].

By using isotope radiolabeled ATP (P^32^ or P^33^) as co-substrate, the phosphorylation reaction can be monitored with high sensitivity in vitro. However, in an in vivo context this approach is not feasible due to the large number of kinases present. Therefore, Shokat and coworkers developed a protein engineering-based approach to enlarge the ATP binding pocket of a specific kinase to accommodate a chemically modified ATP as co-substrate, which would not bind to native kinase enzymes [19]. They engineered the nucleotide binding pocket of the prototypical viral proto-oncogene tyrosine protein kinase Src (v-Src) by mutating the gatekeeper residue Isoleucine at position 338 to Glycine. This point mutation enlarged the binding pocket making the buried region accessible to ATP-competitive analogues with non-polar substituents at the N6 position of the adenine base. The ATP analogue preferentially used by the engineered v-Src kinase as phosphodonor was N^6^-benzyl-adenosine-5′-triphosphate (N6-(benzyl) ATP). The use of γ-phosphate radiolabeled [γ-^32^P] N6-(benzyl) ATP resulted in the v-Src substrates being specifically radiolabeled and identified in the presence of other protein kinases and all other kinase substrates [13, 20]. This approach allowed the identification of cofilin and calumenin as specific v-Src substrates [21]. The conservation of the ATP binding site between different protein kinases makes the approach widely applicable for identifying specific kinase substrates. The gatekeeper residue is identified by the sequence alignment of the kinase of interest with v-Src. In a similar approach, other kinases were engineered to bind specifically modified inhibitors [22–28]. One of the challenges in applying this method to other kinase systems is to identify the optimal combination of kinase binding pocket mutations and ATP derivatives such that the ATP analogue acts as substrate for the engineered, but not the native or other cellular kinases. The mutation should modify size and shape of the ATP binding pocket while the engineered kinases have to remain catalytically active. The ATP analogue has to bind to the engineered kinase at sufficient affinity and in a suitable geometry to accomplish its role as phosphodonor. It needs to enter the engineered binding site, provide the γ-phosphate and leave the binding site in order to allow the engineered protein to perform catalysis. An ATP analogue bound too tight or in the wrong geometry would decrease or abolish the activity of the engineered enzyme.

In this study, we developed a computational protocol that evaluates how mutations within the ATP binding site of protein kinases influence the accommodation of various ATP analogues. The protocol explores pairings of potential mutations and ligand analogues by identifying which residues within the binding pocket could be mutated to accommodate a specific ATP analogue. We tested the protocol on data for different protein kinases from the scientific literature where the Shokat’s method was applied to mutate the gatekeeper position.

## Methods

### Computational protocol

**Fig. 2:**
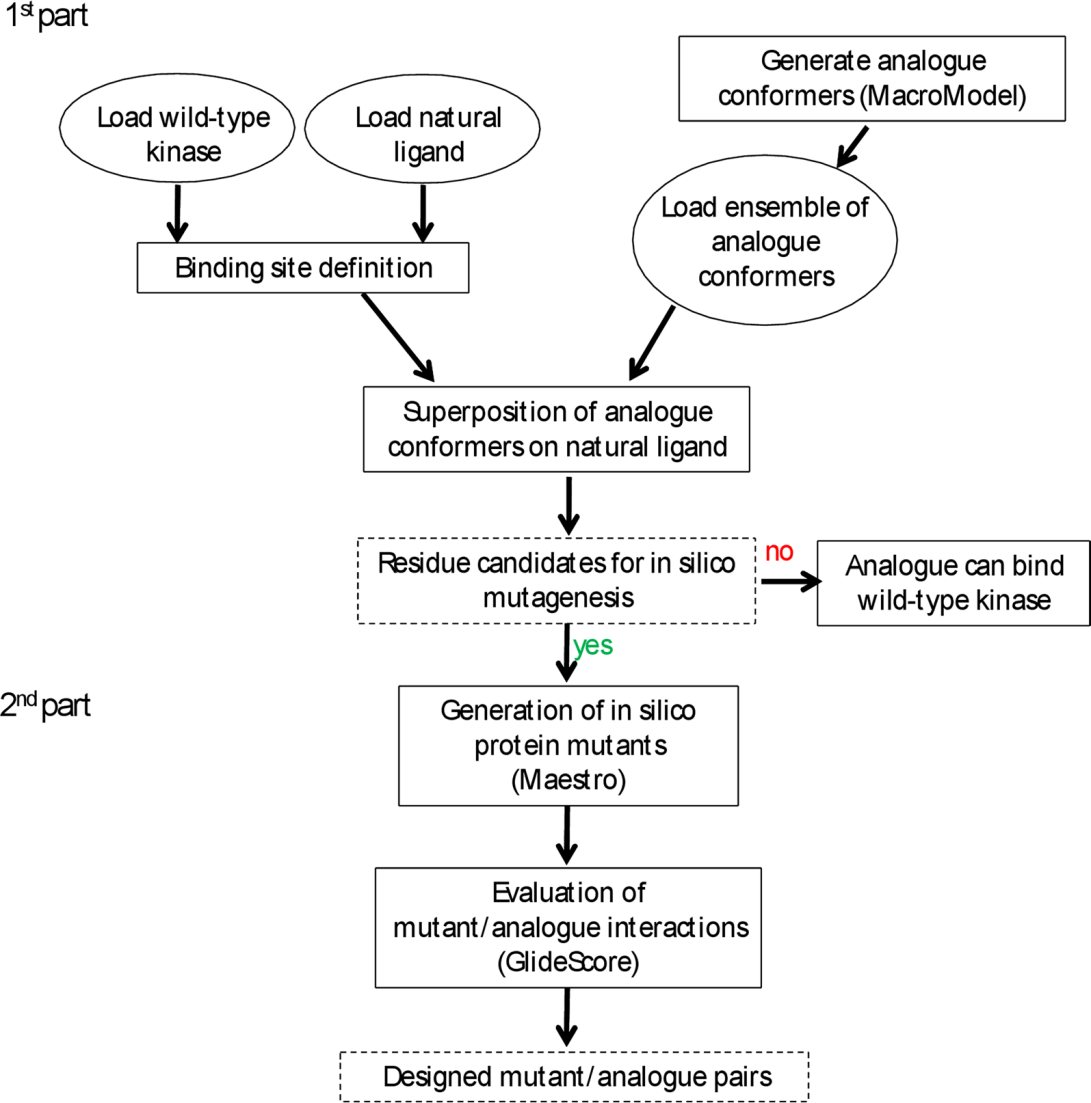
Workflow of the computational protocol. The protocol is organized in two parts, the first part identifies residues to mutate and the 2nd part evaluates mutant-analogue interactions. The specific inputs are depicted in *circles*, steps of the workflow are shown in *rectangles* and outputs are depicted in *rectangles with dashed lines*. In case all analogue conformations are scored as having favorable interactions with the wild type, the analogue is considered to act as substrate for the wild-type protein and thus not further considered

**Fig. 3:**
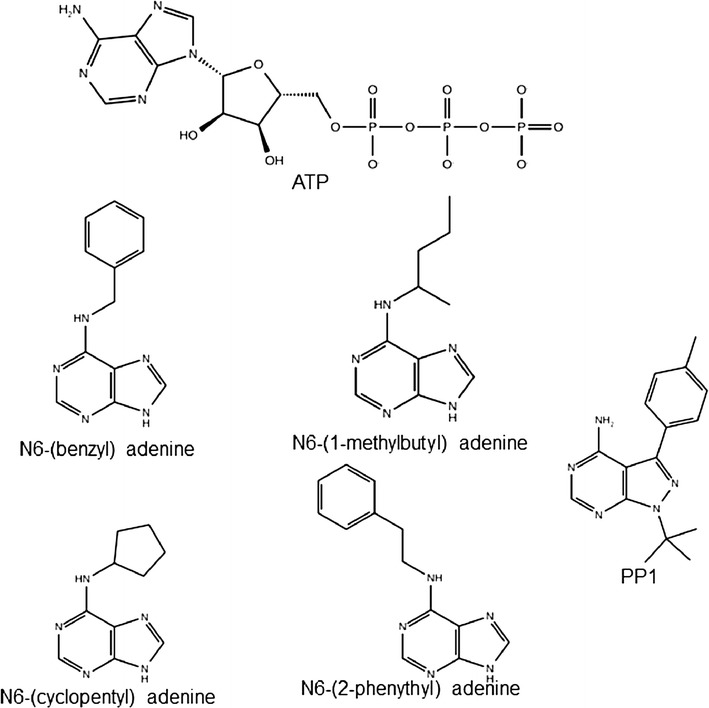
Chemical structures of ATP and ATP-competitive analogues used in this study. For N6-(substituent) ATPs only the structures of the adenine ring and the hydrophobic groups are shown

The computational protocol is organized in two main parts (Fig. 2). Computational models of ligand analogues (N6-(benzyl) ATP, N^6^-(1methylbutyl)adenosine-5′-triphosphate (N6-(1-methylbutyl) ATP), N^6^-cyclopentyl-adenosine-5′-triphosphate (N6-(cyclopentyl) ATP), N^6^-(2-phenythyl)adenosine-5′-triphosphate (N6-(2-phenythyl) ATP), and 1-tert-butyl-3-(4-methylphenyl)-1H-pyrazolo[3,4-d]pyrimidin-4-amine (PP1); Fig. 3) were modelled in Maestro (version 9.5, Schrödinger, LLC, New York, NY, 2013). For each molecule, an ensemble of low energy conformers was generated by performing an in vacuo conformational search keeping the adenine base, the ribose ring, the phosphates and the pyrazolopyrimidine core of PP1 fixed and allowing the bonds of each substituent group to rotate freely. We used the Monte Carlo multiple minimum (MCMM) method [29] for 10,000 steps and OPLS_2005 as force field [30, 31]. During the conformational search, new structures generated were retained if they exhibited conformational energies lower than 100 kJ/mol. The conformation energy cutoff was chosen at 100 kJ/mol to allow for the various geometric approximations made in the force field. It serves as a proxy for the estimated protein–ligand interaction energy. To obtain an ensemble of non-redundant conformations, each conformer was compared with the previous ones and only retained if the root mean square deviation (all atoms) exceeds 0.5 Å. The conformational search was performed with the MacroModel module implemented in the Schrödinger suite (version 10.1, Schrödinger, LLC, New York, NY, USA, 2013).

For each analogue, the ensemble was superposed onto the adenine moiety of the native ATP ligand within the binding pocket of the reference protein. If the distance between an atom of a protein residue and any atom of the substituent group of a ligand analogue in the ensemble is shorter than the sum of their van der Waals [32] radii, the corresponding residue is considered a potential candidate for single-point mutagenesis. If no residues were identified by this approach, the analogue was considered to act as substrate for the native target and thus not further considered. The method was implemented in Python 2.5.4 and contains functions from the OpenStructure software framework [33].

In the second step, the interaction between potential protein mutants and ligand analogues was evaluated using a protein–ligand scoring function. Amino acids at positions identified in the first step were replaced in silico to generate mutant proteins. When a residue was changed into Gly or Ala, the entire structure was relaxed by a minimization step performed using OPLS_2005 as force field in Maestro [34]. When a residue was mutated into an amino acid with a larger side chain, such as Met or Thr, a rotamer scan was performed to identify the most probable rotamer state using Rapid Torsion Scan tool available in Maestro. The kinase mutant-ligand conformer pairs were evaluated and ranked by the protein–ligand scoring function GlideScore [35]. The kinase mutant-ligand conformer structure with the lowest GlideScore was selected and the corresponding Glide energy was computed. The Glide energy is the sum of the Coulomb and van der Waals terms and represents an estimate for the protein–ligand interaction energy. Typically, predicted energies of interaction (Glide energies) correlate better with protein–ligand binding affinities or experimental IC_50_ values than GlideScore [36]. We arbitrarily limited all positive energies to zero as we were only interested in identifying favorable interactions. In the case of engineered kinases and ATP analogue pairs, only the adenine base and the substituent group were scored by GlideScore.

### Kinase data set

A set of 7 protein kinases and 15 mutants for which experimental data were available in literature was used as test set (Table 1). Unless stated otherwise, in silico mutagenesis was performed using Maestro and the structure was prepared with the Protein Preparation Wizard tool [34]. Residues are numbered as as in PDB structures. The crystal structure of JNK bound to ANP (an ATP analogue with an amino group in place of the oxygen between the β and γ phosphates that mimics the natural cofactor) and Mg^2+^ was solved in 1998 (*Homo sapiens*, PDB:1JNK, resolution 2.30 Å, [37]). The crystal structure was prepared for molecular modelling by adding hydrogen atoms, optimizing the hydrogen bonding network, the orientation of the amide groups of Asn and Gln, and the orientation and protonation state of the imidazole ring of His. This optimization allowed for improving interactions between charged groups as well as hydrogen bonds within the structure. The optimization was performed at pH of 7. Finally, a minimization step was applied to relax the entire structure. OPLS_2005 was used as force field and the termination criterion was based on the rmsd of the heavy atoms relative to their initial location (rmsd less than or equal to 0.30 Å). The M108GL168A mutant was obtained by in silico replacing Met108 to Gly and Leu168 to Ala and the structure was prepared as described above.

**Table 1:** Substrate phosphorylation by ATP, kcat/Km, IC_50_ and predicted interaction energy for protein–ligand pairs

Kinases	Ligands	Experimental data	Predicted interaction energies (kcal/mol)
*JNK kinase*		*% Substrate phosphorylation*	
JNK	N6-(benzyl)	99	0^a^
	N6-(2-phenythyl)	98	0^a^
	N6-(cyclopentyl)	97	0^a^
	N6-(1-methylbutyl)	93	0^a^
JNKM108GL168A	N6-(benzyl)	62	−17.34
	N6-(cyclopentyl)	59	−14.42
	N6-(1-methylbutyl)	47	−20.42
	N6-(2-phenythyl)	8	−33.0
*v-Src tyrosine kinase*		*kcat/Km (min* ^*−1*^ *M* ^*−1*^ *)*	
v-Src	ATP	1.6*10^5^	−21.35
v-SrcI338A		1.4*10^4^	−19.91
v-SrcI338G		1*10^4^	−19.01
v-Src	N6-(benzyl) ATP	0	0^a^
v-SrcI338A		2.5*10^4^	−14.92
v-SrcI338G		4.0*10^4^	−29.17
*Tyrosine and serine/threonine kinases*		*IC*_*50*_ * (μM)*	
v-Src	PP1	5 ± 2	0^a^
v-SrcI338F		8 ± 2	0^a^
v-SrcI338 M		8 ± 1	0^a^
v-SrcI338S		0.4 ± 0.05	−28.78
v-SrcI338T		0.1 ± 0.02	−33.32
v-SrcI338 V		0.1 ± 0.02	−27.41
v-SrcI338C		0.07 ± 0.02	−27.44
v-SrcI338A		0.005 ± 0.002	−39.26
v-SrcI338G		0.005 ± 0.002	−38.56
Fyn		0.05 ± 0.02	−36.81
FynT339A		0.005 ± 0.002	−36.21
Abl		0.3 ± 0.03	−32.93
AblT334A		0.03 ± 0.005	−33.86
CamKII		80 ± 10	0^a^
CamKIIF89G		0.5 ± 0.1	−15.74
Cdk2		50 ± 10	0^a^
Cdk2F80G		0.16 ± 0.03	−24.85
P38		0.82 ± 0.2	−34.61
P38T106A		0.0027 ± 0.005	−33.43
P38T106G		0.0027 ± 0.005	−32.78

^a^Interaction energies of 0 kcal/mol represent positive interaction energies

The kinase domain of v-Src differs from that of the cellular protein kinase c-Src at position 338 within the binding pocket (Ile338 in v-Src and Thr338 in c-Src). The crystal structure of c-Src in complex with ANP has been solved (*Homo sapiens*, PDB:2SRC, resolution 1.50 Å, [38]). To obtain a model of v-Src bound to its natural cofactor, we substituted in silico Thr338 into Ile. The v-SrcI338A and v-SrcI338G mutants were obtained in the same way.

To obtain a model of v-Src in complex with a pyrazolopyrimidine inhibitor, PP1, the structure of v-Src bound to ANP was superposed onto the structure of the hematopoietic cell kinase (Hck, a homologous protein) in complex with PP1 (*Homo sapiens*, PDB:1QCF, resolution 2.00 Å, [39]). The superposition was based on residues belonging to the hinge regions (residues 338–341 in both v-Src and Hck). The coordinates of PP1 were copied into the v-Src binding site and the complex was then prepared and minimized as described before. The same procedure was used for all other protein kinases and mutants studied in the same paper, proto-oncogene c-Fyn (Fyn, *Homo sapiens*, PDB:2DQ7, resolution 2.80 Å, [40]), abelson murine leukemia viral oncogene homolog 1 (Abl, *Homo sapiens*, PDB:2G1T, chain D, resolution 1.80 Å, [41]), calcium/calmodulin-dependent protein kinase type II subunit alpha (CamKII, *Homo sapiens*, PDB: 2VZ6, chain B, resolution 2.30 Å, [42]), cyclin-dependent kinase 2 (Cdk2, *Homo sapiens*, PDB:1HCK, resolution 1.90 Å, [43]), and mitogen-activated protein kinase p38 alpha (P38, *Homo sapiens*, PDB:1DI9, resolution 2.60 Å, [44]).

The complex of Fyn bound to the PP1 conformer with the best GlideScore was minimized *in vacuo* without constraints. We used the Polak-Ribier Conjugate Gradient (PRCG) as method for 2500 steps [45]. The same procedure was used for the complexes of FynT339A, Abl and AblT334A. The procedure was performed using MacroModel.

### Data comparison

All plots reported in this paper were made using the Matplotlib [46] and NumPy packages [47]. In the plot of JNKM108GL168A, the interaction energies were scaled between 0 and 100 to fit the same range of observed phosphorylation values (expressed as percentage of phosphorylation). The lowest Glide energy was set to 0 and the highest to 100. The plots of v-Src, v-SrcI338A and v-SrcI338G in complex with ATP and N6-(benzyl) ATP were created by comparing the experimental catalytic efficiency (kcat/Km) and the predicted interaction energies (Glide energies). To correlate experimental and predicted data, we computed the negative logarithm of the kcat/Km ratio. The plots of tyrosine kinases and serine/threonine kinases in complex with PP1 were made measuring the linear correlation between the predicted interaction energies and the experimental measured pIC_50_ (−log(IC_50_)). For each family, the Pearson correlation coefficient was computed.

## Results and discussion

**Fig. 4:**
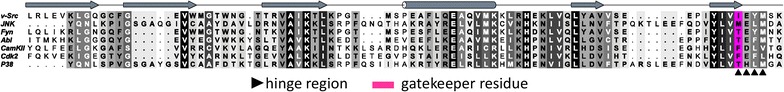
Sequence alignment of the N-lobe and hinge region of the seven wild-type protein kinases belonging to our data set. The alignment is build using the T-Coffee web server [52]. Residues are colored by percentage of identity. Secondary structure elements are represented as follows: β strands as *arrows*, α helixes as *cylinders*, and coils as *lines*

The gatekeeper position in protein kinases controls the accessibility to a buried region at the end of the ATP binding pocket. Shokat has demonstrated that by mutating the gatekeeper residue, the size and shape of the ATP binding site can be modified such that the engineered kinases can use specific chemically modified ATP molecules as co-substrates. The gatekeeper residues of the kinases in our test set equivalent to position Ile338 in v-Src are shown in Fig. 4. Kinases with large gatekeeper residues, such as Ile or Met, do not allow for binding of ligand analogues with bulky side chains (e.g. v-Src or JNK) whereas those with smaller gatekeeper residues, e.g. Thr, can accommodate analogues within the binding pocket (for instance Fyn or Abl).

We tested the performance of our computational protocol on a data set containing 7 wild-type protein kinases and 15 mutants (Table 1). The ATP-competitive ligands used in the test set are N6-(substituent) ATPs with bulky hydrophobic groups at the N6 position of the adenine ring and the pyrazolopyrimidine PP1 (Fig. 3). The pyrazolopyrimidine core of PP1 mimics the adenine ring of ATP in binding within the nucleotide pocket [39]. The proteins belonging to the data set are from three independent experimental studies where Shokat’s method was applied and tested. For JNK, the ability of the ATP-competitive ligands to bind kinase mutants was tested by measuring their ability to inhibit the phosphorylation of a given substrate in presence of ATP (% substrate phosphorylation) [26]. For v-Src, the kinetic efficiency (kcat/Km) was used to measure the preference of protein kinases and/or mutants for different co-substrates [20]. For kinases belonging to tyrosine and serine/threonine families, the potency of PP1 to inhibit protein kinases and/or mutants (IC_50_) was measured [48]. We applied our computational approach to identify residues to mutate within the ATP binding pocket of these protein kinases, and the predicted protein–ligand interaction energies (Glide energies) were then compared to the published experimental data.

### JNK and N6-(substituted) ATPs

Habelhah and coworkers modified the JNK ATP binding site so that it binds N6-(substituted) ATPs that cannot be accommodated by the wild-type binding pocket. The designed JNK mutant-ATP analogue pair allowed for the identification of novel JNK substrates [26]. To determine the ATP analogue with the highest affinity for the engineered JNK, they compared four N6-(substituent) ATP analogues. Their efficiency as phosphodonor was tested by measuring their ability to prevent phosphorylation of substrates by ATP when they are added in excess with respect to ATP. For wild-type JNK and the ATP analogues the percentage of substrate phosphorylation ranged from 99 to 93%, showing the inability of the wild-type kinase to accommodate any of the four ATP analogues. On the other hand, the JNKM108GL168A mutant was able to accommodate N6-(substituent) ATPs and N6-(2-phenythyl) was the ligand with the highest affinity to the mutant (the percentage of substrate phosphorylation is 8%) (Table 1).

**Fig. 5:**
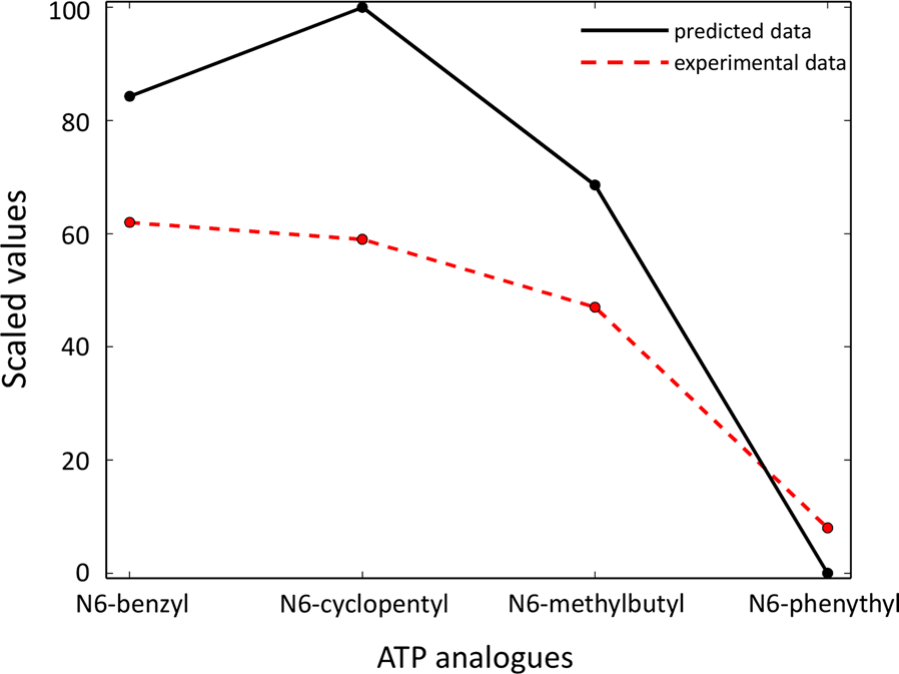
Comparison of experimental and predicted data for engineered JNKM108GL168A and ATP analogues. Plot shows the percentage of substrate phosphorylation by ATP in presence of ATP analogues and the scaled predicted interaction energies for the engineered JNK and the four ATP analogues. The percentage of phosphorylation and the predicted binding energies scaled between 0 and 100 are shown for the different ATP analogues

We applied the computational protocol to JNK and the four N6-(substituent) ATPs. For the wild-type we could not identify a low energy binding conformation without steric hindrance, indicating that none of the ATP analogues can fit into the wild-type JNK ligand binding pocket (Table 1). The computational protocol identified two residues within the JNK binding site as potential candidates for double mutagenesis in order to enlarge the binding pocket, the gatekeeper Met108 and Leu168. We in silico replaced them with Gly and Ala, respectively, and evaluated the interaction of the engineered JNK with each ATP analogue. The complex of JNKM108GL168A and N6-(2-phenythyl) ATP shows the lowest Glide energy, implying that N6-(2-phenythyl) ATP is the substrate with the best ability to bind the engineered JNK in a constructive manner (Table 1). Employing our computational protocol, in first instance we reproduce the experimental findings that identify Met108 and Leu168 as amino acids to mutate within the JNK binding pocket in order to enlarge it. Furthermore, we correctly reproduce the relative ranking of the four ATP analogues as substrates for the engineered JNK classifying N6-(2-phenythyl) ATP as the best substrate (Fig. 5).

### v-Src and N6-(benzyl) ATP

Shokat and coworkers engineered v-Src to produce a kinase mutant that preferentially used N6-(benzyl) ATP as co-substrate instead of the natural nucleotide (ATP) [20]. They performed kinetic measurements revealing that wild-type v-Src had a substrate preference for ATP over the ATP analogue (1.6*10^5^ min^−1^ M^−1^ vs 0) and the I338G mutant preferentially used N6-(benzyl) ATP as co-substrate over the natural ATP (the kcat/Km ratio is 4–1).

**Fig. 6:**
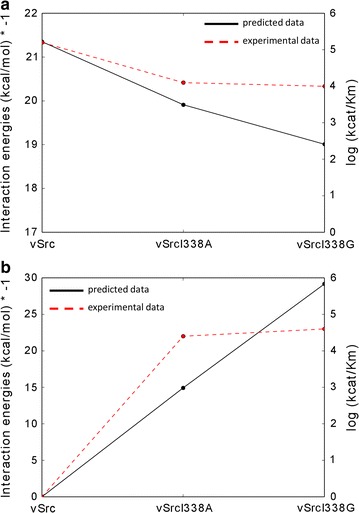
Comparison of the catalytic efficiency and predicted interaction energy for v-Src, v-SrcI338A, and v-SrcI338G with ATP (**a**) and with N6-(benzyl) ATP (**b**). Plots show the trend of the kcat/Km ratio and the predicted interaction. Shown on the *x-axis* are the wild-type protein and the two mutants. The primary *y-axis* (on the *left*) is the predicted negative interaction energies and the secondary *y-axis* (on the *right*) is the log of the kcat/Km ratio

We used our approach and identified the gatekeeper Ile338 as being a good candidate for point mutation to enlarge the v-Src ligand-binding site, in agreement with Shokat’s experimental findings. We scored mutant models I338A and I338G in complex with N6-(benzyl) ATP and both had negative energy of interaction with the ATP analogue implying their ability to accommodate it within their engineered binding pocket. The predicted interaction energies well reproduced the trend of the experimental kinetic constants (Table 1). Wild-type v-Src, v-SrcI338A and v-SrcI338G are able to interact with ATP with almost equal interaction energies (Fig. 6a). Wild-type v-Src cannot accommodate N6-(benzyl) ATP because of the steric overlaps between the side chain of Ile338 and the benzyl group attached at the N6 position of the ATP analogue. V-SrcI338A and v-SrcI338G have enlarged binding pockets that accommodate the ATP analogue in a constructive interaction. V-SrcI338G has the best predicted energy of interaction and is confirmed as the best binder to the ATP analogue (Fig. 6b).

### Tyrosine and serine/threonine protein kinases and PP1

A study conducted by Liu and coworkers analyzed how the gatekeeper residue controls the ability of PP1 to inhibit protein kinases [48]. The gatekeeper amino acid corresponds to Ile338 in v-Src, Thr339 in Fyn, Thr334 in Abl, Phe89 in CamKII, Phe80 inCdk2, and Thr106 in P38. The study showed that residues equal to or larger than Ile, such as Phe and Met, make PP1 a less potent inhibitor (IC_50_ ≥ 1 μM) whereas residues smaller than Ile, such as Ser, Thr, Val, Cys and especially Ala and Gly increase the potency of PP1 (IC_50_ values ranging from 0.05 to 0.82 μM).

**Fig. 7:**
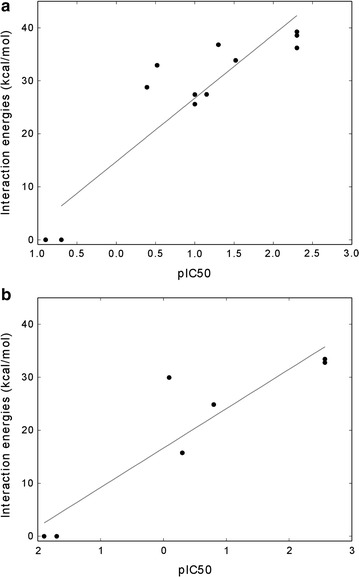
Correlation plots of the predicted interaction energies versus the experimental pIC_50_. **a** Src family tyrosine kinase with PP1. The correlation coefficient is 0.85. **b** Serine/threonine kinases with PP1. The correlation coefficient is 0.75. Data from Table 1

We mutated the gatekeeper residues to obtain structural models of the engineered kinases and analyzed the correlation between predicted energies of interaction of wild-type and engineered kinases with PP1 and inhibition data (IC_50_). For both tyrosine kinase and serine/threonine kinase families the predicted interaction energies reproduced the trend of the inhibitor potency (Table 1). A positive correlation between the experimental −log(IC_50_) (pIC_50_) and the predicted interaction energies was found for both families, with a Pearson correlation of 0.85 for the Src tyrosine kinases and of 0.75 for the serine/threonine kinases (Fig. 7). Our computational protocol discriminated well between protein variants that are inhibited by PP1 (negative interaction energies, e.g. v-SrcI338S or CamKIIF89G) and proteins that are not inhibited (positive interaction energies, e.g. v-Src, v-SrcI338F or Cdk2). In the specific case of v-Src, the protocol is able to reproduce the ranking of the mutants and identify which engineered kinases are the best binders to PP1, with v-SrcI338A and v-SrcI338G being identified as the best in agreement with IC_50_ values (Table 1). Despite the overall good correlation between inhibition data and predicted interaction energies, in some cases GlideScore does not discriminate between a good and very good binder to PP1, such as wild-type Fyn and FynT339A or wild-type Abl and FynT334A. Threonines within the binding sites of wild-type Fyn and Abl allow the binding of PP1 with an IC_50_ of 0.05 and 0.3 μM, respectively. The mutagenesis of Thr into the smaller Ala results, in both cases, in an increase of the IC_50_ by a factor of 10 (from 0.05 to 0.005 μM for Fyn and from 0.3 to 0.03 μM for Abl). The predicted interaction energies do not mirror that increase. For Fyn and FynT339A the predicted energies of interaction with PP1 are almost the same, 36.81 and 36.21 kcal/mol, respectively and the same result is obtained for Abl and AblT334A in complex with PP1 with interaction energies of 32.93 and 33.86 kcal/mol, respectively.

We explored to which extend energy minimization of the complex models before scoring would lead to better correlation between experimental and predicted data. For both Fyn and Abl and the respective mutants we considered the protein-PP1 complexes with the best GlideScore and minimize them without constraints. Although the introduction of a minimization step results in lower predicted protein-inhibitor interaction energies (Table 2), GlideScore was not capable of differentiating relative affinity between generally strong protein-inhibitor interactions. The use of scoring functions more sensitive to the subtle changes in protein–ligand interactions, or scoring functions tailored to specific binding site properties [49] might help to overcome the inability of GlideScore in discriminating relative binding affinity for good binders.

**Table 2:** IC_50_ and predicted energies computed before and after minimization for four kinase-PP1 complexes

Protein-PP1 complexes	IC_50_ (μM)	Predicted interaction energies (kcal/mol)	Predicted interaction energies after minimization (kcal/mol)
Fyn-PP1	0.05 ± 0.02	−36.81	−44.30
FynT339A-PP1	0.005 ± 0.002	−36.21	−45.83
Abl-PP1	0.3 ± 0.03	−32.93	−43.54
AblT334A-PP1	0.03 ± 0.005	−33.86	−42.95

The main goal of this study is to identify, which binding-site residues of the target kinase could be mutated to accommodate a specific ATP analogue as co-substrate without interfere with the catalytic activity of the kinase protein. To reach this goal, we used a protein structure derived by X-ray crystallography in complex with the natural ATP substrate as starting point. In order to be able to act as co-substrate in catalysis, a ligand was assumed to be able to bind in place of the natural substrate in a low-energy conformation. We therefore modelled each modified ATP with adenine, ribose and phosphates geometry identical to the native ATP within the kinase binding site, and sampled the conformational ensemble of substituents for low energy conformations which could be accommodated in the binding site. Our computational approach reproduces the experimental data available in literature. The method is able to discriminate between residues that have to be mutated into smaller ones to allow the accommodation of ligand analogues, (e.g. Ile338 in v-Src) and residues that instead allow for the binding of specific analogues within the wild type enzyme (e.g. Thr339 of Fyn).

Shokat and coworkers tested 12 N6-(substituent) ATPs with 7 v-Src mutants in order to identify the optimal combination of a mutation within the v-Src ligand-binding pocket and a chemical derivative of ATP to use for identifying the specific v-Src substrates [19, 20], and identified N6-(benzyl) ATP as suitable substrate for an engineered v-Src with an enlarged binding pocket, v-SrcI338G. Their approach was based on the ‘bump-and-hole’ model [50, 51]. The gatekeeper residue was mutated into a small amino acid generating a ‘hole’ within the ligand-binding site that can accept ligands with bulky substituent groups, ‘bumps’. The method was based on exploring shape complementarity between the enlarged kinase binding pocket and the ATP derivative.

The computational protocol we developed in this work can help to rationalize the experimental procedure to identify the substrates of a specific kinase: It aims to prescreen a large number of computationally modelled mutant-analogue complexes, in order to reduce the number of pairs to test in vitro and/or in vivo. Furthermore, in our procedure the gatekeeper position could be replaced into each of the other 19 amino acids. This would allow identifying new residues for mutation based on shape complementarity as well as specific protein–ligand interactions between side chains of mutated residues and substituent groups of ATP analogues.

## Conclusions

We developed a computational protocol for evaluating how mutations at the gatekeeper position influence the accessibility of ATP-competitive ligands within the binding site of kinase mutants. Shokat and coworkers have experimentally identified the gatekeeper position as suitable for engineering kinases with modified co-substrate specificity. Our computational protocol allows further exploration of this approach via two routes. The first route is able to provide a relative rank of various ATP analogues for a given gatekeeper residue mutation. The second route provides a way to evaluate for given ligand analogue, which mutations at the gatekeeper residue position would be compatible. The computational screen of a large ensemble of potential mutant-analogue pairs can reduce the number of experimental essays to perform resulting in a significant reduction of the time and the cost of the whole experiment. Besides protein–ligand shape complementarity, our computational protocol allows the evaluation of different types of interactions between an engineered kinase and an ATP derivative. This will allow exploring gatekeeper mutations exhibiting specific polar interactions with the ATP analog, which have not yet been explored in the literature.

## Abbreviations

Abl: abelson murine leukemia viral oncogene homolog 1
ALK: anaplastic lymphoma kinase
ANP: phosphoaminophosphonic acid-adenylate ester
ATP: adenosine triphosphate
c-Fyn: proto-oncogene c-Fyn
JAK3: Janus Kinase 3
MCMM: Monte Carlo multiple minimum
P38: mitogen-activated protein kinase p38 alpha
PDB: Protein Data Bank
PP1: 1-tert-butyl-3-(4-methylphenyl)-1H-pyrazolo[3,4-d]pyrimidin-4-amine
v-Src: viral proto-oncogene tyrosine protein kinase Src
